# Relationships between early age at onset of psychotic symptoms and treatment resistant schizophrenia

**DOI:** 10.1111/eip.13174

**Published:** 2021-05-16

**Authors:** Felice Iasevoli, Eugenio Razzino, Benedetta Altavilla, Camilla Avagliano, Annarita Barone, Mariateresa Ciccarelli, Luigi D'Ambrosio, Marta Matrone, Federica Milandri, Danilo Notar Francesco, Michele Fornaro, Andrea de Bartolomeis

**Affiliations:** ^1^ Section of Psychiatry ‐ Unit on Treatment Resistant Psychosis, and Laboratory of Molecular and Translational Psychiatry, Department of Neuroscience University School of Medicine Federico II Naples Italy

**Keywords:** antipsychotic, clozapine, psychosis, refractory

## Abstract

**Aim:**

Early age at schizophrenia onset (EOS) has been associated with a worse clinical course, although previous studies reported substantial heterogeneity. Despite the relevance of the subject, the relationship between the age of onset and treatment resistant schizophrenia (TRS) is less clear.

**Methods:**

We screened 197 non‐affective psychotic patients. Of these, 99 suffered from schizophrenia and were putative TRS and were included in a prospective 4‐to‐8‐week trial to assess their response to antipsychotics. According to status (TRS/nonTRS) and age‐at‐onset (early: ≤18 years, EOS; adult: >18 years, adult onset schizophrenia [AOS]) patients were subdivided in EOS‐TRS, EOS‐nonTRS, AOS‐TRS, AOS‐nonTRS. Multiple clinical variables were measured and compared by analysis of covariance (ANCOVA), using age as a covariate. Two‐way analysis of variance (ANOVA) was used to assess whether significant differences were attributable to TRS status or age‐at‐onset.

**Results:**

The rate of TRS patients was significantly higher in EOS compared to AOS. At the ANCOVA, EOS‐TRS had significantly worse clinical, cognitive, and psychosocial outcomes compared to the other groups. Overall, EOS‐TRS were more impaired than EOS‐nonTRS, while significant differences with AOS‐TRS were less consistent, albeit appreciable. Two‐way ANOVA demonstrated that, in the majority of the investigated variables, the significant differences among groups were attributable to the TRS status effect rather than to age‐at‐onset or combined effects.

**Conclusions:**

These results suggest that refractoriness to antipsychotics may be strongly linked to the early onset of psychotic symptoms, possibly as a result of common neurobiology.

## 1 INTRODUCTION

Schizophrenia is a chronic psychiatric disorder affecting ~0.3% of the world population (Charlson et al., 2018) and characterized by pleomorphic symptomatology (Howes & Murray, 2014). Although the typical age at onset for psychotic symptoms of schizophrenia is in early adulthood (adult onset schizophrenia, AOS) (Ochoa et al., 2012), acute psychotic symptoms may already occur in childhood or adolescence, before the age of 18. This condition is known as early onset schizophrenia (EOS) (Clemmensen et al., 2012) and accounts for 40% of the total number of cases (Hollis & Rapoport, 2011). Patients affected by EOS show signs of more severe neurodevelopment alterations (Nicolson & Rapoport, 1999) and display an increased genetic vulnerability (Ahn et al., 2016). According to this putative genetic and neurobiological background, EOS has been associated with worse prognosis, greater symptom severity, social withdrawal, poorer cognitive performances (Clemmensen et al., 2012; Rabinowitz et al., 2006; Sato et al., 2004).

Treatment resistant schizophrenia (TRS) is defined as the persistence of significant symptoms despite at least two adequate treatment trials, each for a minimum duration and dosage (Howes et al., 2017; Kane et al., 1988, 2019). Compared to schizophrenia patients who respond to antipsychotic agents (herein: non‐TRS), patients affected by TRS display worse cognitive functioning (de Bartolomeis et al., 2013; Frydecka et al., 2016; Iasevoli et al., 2013), more dysfunctional social achievements and disturbed functional capacity (Iasevoli et al., 2016; Iasevoli, D'Ambrosio, et al., 2018b), higher prevalence of Neurological Soft Signs (de Bartolomeis et al., 2018), unique course of brain structure and function (Harvey & Rosenthal, 2016). Notably, early age at onset has been regarded as a risk factor of developing resistance to antipsychotic treatments (Iasevoli et al., 2016; Lally et al., 2016).

EOS and TRS patients may represent specific subsets of schizophrenia subjects, both affected by greater severity of symptoms and cognitive deficits of presumed neurodevelopmental origin (Chen et al., 2019; de Bartolomeis et al., 2018; Harvey & Isner, 2020; Iasevoli, Avagliano, et al., 2018a). Also, these subtypes may share a distinctive neurobiology involving non‐dopaminergic mechanisms (Demjaha et al., 2014; Fachim et al., 2019; Jauhar et al., 2019; Leung et al., 2019; Mouchlianitis et al., 2016; Nucifora et al., 2019). However, to date, no studies have investigated whether EOS TRS patients differ in clinical outcomes from EOS non‐TRS patients and AOS patients. The objective of this study is to evaluate the hypothesis that EOS TRS patients have more severe clinical course, more impaired cognitive performances, and poorer social outcomes than both EOS non‐TRS and AOS patients.

## 2 PATIENTS AND METHODS

### 2.1 Study design

Part of the patients included in the present sample belonged to the sample recruited for previously published reports (de Bartolomeis et al., 2018; Iasevoli, D'Ambrosio, et al., 2018b). Additional participants were added in the database compared to the sample of the publications mentioned above, to reach estimated study power.

Participants were from the Outpatient Unit on Treatment Resistant Psychosis, University ‘Federico II’ of Naples, after referral from community due to supposed treatment resistant psychotic symptoms. All patients signed a written informed consent form, approved by our local Ethical Committee. All procedures carried out in the present study complied with the principles laid down by the Declaration of Helsinki, revised Hong Kong 1989.

Eligible patients had to meet all inclusion criteria and none of the exclusion criteria. All consecutive patients meeting criteria for eligibility and conferring consent were recruited. Inclusion criteria were: (i) age within the 18–65 years range; (ii) diagnosis of schizophrenia; (iii) being under antipsychotics; (iv) stabilized symptoms, including persistent psychotic symptoms with no evidence of actual or recent (i.e., in the last 3 months prior assessments) worsening. Exclusion criteria were: (i) intellectual disability (according to DSM‐5 diagnostic criteria); (ii) severe medical diseases (as certified by clinical records, medical examination and/or by ad‐hoc adjunctive analyses); (iii) non‐schizophrenia psychotic disorders (including brief psychotic disorder, schizophreniform disorder, schizoaffective disorder, delusional disorder, schizotypal personality disorder, affective psychosis); (iv) psychotic symptoms due to another medical condition or to substances/medications.

All diagnoses were made by trained psychiatrists through the Structured Interview for Diagnosis (SCID‐I). A total number of 198 patients with psychotic symptoms were screened at the moment of the data analysis. Among these, 99 were included in the final sample.

### 2.2 Prospective trial procedure and response to antipsychotics' assessment

Before starting with the assessment procedures, pseudo‐resistance factors were assessed. Pseudo‐resistance may be defined as the lack of antipsychotic response that cannot be attributed to the inadequacy of pharmacological action of the therapeutic agent, but depended on other modifiable/not modifiable factors, including: (i) lack of compliance; (ii) concomitant substance abuse; (iii) medical disorders or medications affecting antipsychotic pharmacokinetics/pharmacodynamics; (iv) adverse and detrimental psychosocial conditions (Iasevoli et al., 2016). Patients whose psychotic symptoms may be reconducted to pseudo‐resistance were excluded from the sample. Also, operational criteria were used to assess whether history of non‐response was met (Howes et al., 2017; Kane et al., 1988). Patients whose clinical history may not be reliably reconstructed or whose lack of response to antipsychotics was due to pseudo‐resistance were excluded from the eligible sample.

Eligible patients underwent a prospective trial with a new antipsychotic, whose choice was based on the clinician advice. Clozapine was not yet prescribed at this stage. Positive and Negative Syndrome Scale (PANSS) ratings were collected before antipsychotic initiation and during the follow‐up. Since standard PANSS rating could underestimate response, we chose to adopt rescaling (Obermeier et al., 2011). Therefore, we subtracted a basal 30‐point score (corresponding to the minimum score of 1 for each item) to both baseline and all subsequent evaluations.

**FIGURE 1:**
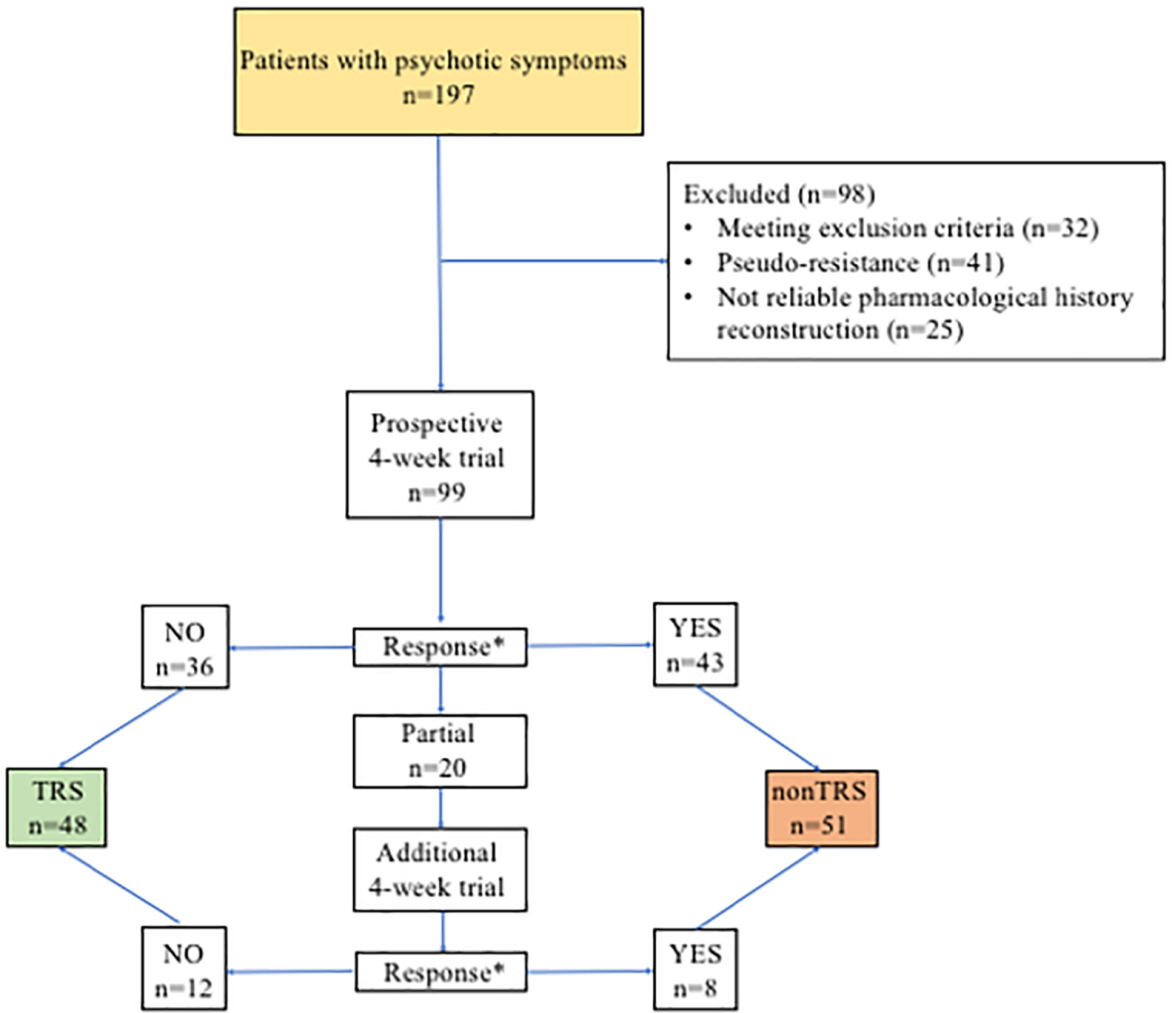
Flowchart of the prospective trial for treatment resistant schizophrenia (TRS) definition. In this flowchart, are reported the procedures to categorize patients as TRS/nonTRS. After controlling for exclusion criteria and pseudo‐resistance factors, putative TRS patients entered a 4‐week prospective antipsychotic trial. The antipsychotic was chosen by the clinician, mostly based on previous efficacy/tolerability history. Positive and Negative Syndrome Scale (PANSS) was administered at baseline and after 4 weeks of antipsychotic treatment. *: Response was defined as at least a 50% reduction of PANSS total score from baseline. Non‐response was defined as less than 25% reduction from baseline, partial response as a >25% and <50% reduction from baseline. Partial responders underwent an additional 4‐week trial, and then again assessed by the PANSS. Non‐responders were categorized as TRS (*n* = 48). Responders were considered nonTRS (*n* = 51). We also tested an alternative TRS definition based on PANSS Positive scale score only, that was derived from the last PANSS administered (i.e., at the end of the first 4‐week prospective trial for full responders and non‐responders; at the end of the second 4‐week prospective trial for partial responders). Responders (*n* = 38) were considered as those having less than three items scoring at least 4 and no item scoring 5 at the PANSS Positive. Non‐responders (*n* = 61) were those still scoring 4 at three items or scoring 5 at one item of the PANSS Positive despite the longitudinal trial with a target antipsychotic dose

Response was defined as the reduction of >50% baseline PANSS score after 4 weeks of antipsychotic at the target therapeutic dose (Leucht et al., 2005). Responder patients were regarded as nonTRS. A reduction <25% indicated non‐response, and therefore these patients were labelled TRS. A PANSS reduction <50% but >25% indicated partial response. Partial responder patients underwent an additional 4‐week trial where antipsychotic doses can be further increased. At the end of this additional trial, PANSS score reduction <50% compared to baseline indicated non‐response (i.e., TRS) (Figure 1).

### 2.3 Assessments

Clinical‐demographic data of the sample were recorded. Antipsychotic doses (CPZ) were transformed in chlorpromazine‐equivalent doses according to previously published papers (Gardner et al., 2010). All assessments were carried out at the end of the prospective trial. Assessments included the Clinical Global Impression‐Severity (CGI‐S), the Specific Level of Functioning (Mucci et al., 2014), and the Personal and Social Performance (PSP) scale (Morosini et al., 2000). All rating scales and cognitive assessments were concluded in a single‐day session, where possible. Neurological soft signs were assessed by the Neurological Evaluation Scale (NES) (Buchanan & Heinrichs, 1989) by two experienced raters. In all cases, inter‐rater reliability was between 0.8 and 0.9.

Patients were assessed for cognitive performances in the following discrete cognitive domains by trained psychiatrists: Sustained and Selective Attention by the Continuous Performance Task (CPT); Verbal Memory by the List Learning task; Visuospatial Memory (VSM) by the Brief VSM test‐Revisited; Working Memory by the Digit Sequencing task; Verbal Fluency by the Category Instances task and the Controlled Oral Word Association test; Problem Solving by the Tower of London task; Speed of Information Processing by the Symbol Coding task. Raw data from each task were adjusted in corrected scores, according to values in the Italian normative population (Anselmetti et al., 2008; Galderisi et al., 2014). High corrected scores corresponded to better preservation of cognitive status.

Administration of the Italian UCSD performance‐based skills assessment (UPSA)‐extended version (Iasevoli, D'Ambrosio, et al., 2018b) was made by trained authors belonging to the staff of the present study.

### 2.4 Definition of EOS and AOS

According to one of the most employed definitions in the literature (Immonen et al., 2017), age at onset was defined as the age at which the first, clinically meaningful positive psychotic symptoms occurred. EOS was defined as the onset of psychotic symptoms at age 18 or prior. Schizophrenia with symptoms onset after age 18 was defined as AOS (Clemmensen et al., 2012).

### 2.5 Alternative PANSS Positive‐based definition of TRS

Antipsychotic agents are first directed at targeting positive symptoms of schizophrenia, while there is no current evidence that they may significantly ameliorate negative symptoms (Krause et al., 2018). Accordingly, we attempted an alternative PANSS Positive Symptom score‐based classification of TRS/nonTRS patients, borrowed by the definition of Prominent Positive Symptoms (Rabinowitz et al., 2013). Patients were considered non‐responders to antipsychotics (thereafter denoted as TRSp) if they had a score ≥4 on at least three items or a score ≥5 on at least one item of the Positive Symptom subscale at the PANSS evaluation carried out at the end of the prospective antipsychotic trial. This alternative definition aimed at minimizing the impact of negative symptoms on the definition of antipsychotic‐refractory schizophrenia.

### 2.6 Statistical analyses and data evaluation

All statistical procedures were carried out using the SPSS 24.0® software. Descriptive statistics were used to report clinical and socio‐demographic data. Independent‐sample Student's *t* and Chi‐square tests were used to compare quantitative and categorical data, respectively, between EOS and AOS. In all tests, significance was set at *p* < 0.05 (two‐tailed). Analysis of covariance (ANCOVA) was used to compare clinical outcomes among groups including age as a covariate. The least significant difference test was used as the post‐hoc test for pairwise comparisons.

Two‐way ANOVA with status (TRS vs. non‐TRS) and age at onset (EOS vs. AOS) as the independent variables was used to evaluate whether the significant differences found at the ANCOVA may depend on independent or combined TRS status/age at onset effects.

All the data in the present study are available on request due to privacy/ethical restrictions.

## 3 RESULTS

### 3.1 Comparisons between EOS and AOS patients

Demographic variables have been listed in Table 1. Among these, age was significantly lower in EOS compared to AOS. Age was then included in all subsequent analyses as a covariate. No significant gender or educational level differences were found. Educational levels were not significantly different even when corrected for age. On their access to our outpatient unit, the participants were under the following antipsychotics: aripiprazole (17.6%); haloperidol (4.9%); olanzapine (12.6%); quetiapine (16.8%); paliperidone (8.4%); perphenazine (6.3%); risperidone (23.1%); ziprasidone (10.3%). After antipsychotic switch to enter the 4‐week prospective trial, the following antipsychotics were prescribed: amisulpride (3.3%); aripiprazole (28.3%); olanzapine (16.4%); paliperidone (12.2%); perphenazine (4.2%); quetiapine (8.8%) risperidone (22.5%); ziprasidone (4.3%).

**TABLE 1:** Demographic and clinical factors

Variable	EOS (*n* = 53)	AOS (*n* = 46)	Statistics	Adjusted statistics^a^	Adjusted statistics^b^
Age	31.7 ± 9.2	41.8 ± 9.9	*t*_(df = 97)_ = −5.2 ***p* < 0.0001**		*F*_(df = 1,96)_ = 120.9 ***p* < 0.0001**
Gender (m/f%)	69.8/30.2	63.0/37.0	*χ*_(df = 1)_ = 0.51 *p* = 0.52		
Years of schooling	12.3 ± 2.4	12.4 ± 3.4	*t*_(df = 97)_ = −0.2 *p* = 0.85	*F*_(df = 1,96)_ = 0.5 *p* = 0.46	*F*_(df = 1,96)_ = 0.25 *p* = 0.86
Age at onset	16.6 ± 3.09	26.9 ± 5.9	*t*_(df = 96)_ = 10.96 ***p* < 0.0001**	*F*_(df = 1,96)_ = 74.37 ***p* < 0.0001**	*F*_(df = 1,96)_ = 121.6 ***p* < 0.0001**
AaFP	18.7 ± 4.2	28.2 ± 6.2	*t*_(df = 97)_ = −8.9 ***p* < 0.0001**	*F*_(df = 1,96)_ = 45.02 ***p* < 0.0001**	*F*_(df = 1,96)_ = 78.6 ***p* < 0.0001**
PANSS total	93.8 ± 17.9	86.7 ± 14.1	*t*_(df = 97)_ = 2.2 ***p* = 0.03**	*F*_(df = 1,96)_ = 5.6 ***p* = 0.02**	*F*_(df = 1,96)_ = 4.5 ***p* = 0.03**
PANSS General psychopathology	49 ± 9.9	45 ± 7.8	*t*_(df = 97)_ = 2.2 ***p* = 0.03**	*F*_(df = 1,96)_ = 6.4 ***p* = 0.01**	*F*_(df = 1,96)_ = 4.9 ***p* = 0.02**
Disorganization factor	31.8 ± 7.7	28.5 ± 5.3	*t*_(df = 92)_ = 2.4 ***p* = 0.02**	*F*_(df = 1,91)_ = 7.5 ***p* = 0.007**	*F*_(df = 1,91)_ = 5.2 ***p* = 0.02**
NES total	20.6 ± 12	18.2 ± 8.3	*t*_(df = 97)_ = 1.1 *p* = 0.25	*F*_(df = 1,96)_ = 5.4 ***p* = 0.02**	*F*_(df = 1,96)_ = 1.2 *p* = 0.26
Visuospatial memory	27.4 ± 16.4	35.2 ± 13	*t*_(df = 72)_ = −2.3 ***p* = 0.03**	*F*_(df = 1,71)_ = 7.1 ***p* = 0.01**	*F*_(df = 1,71)_ = 4.7 ***p* = 0.03**
UPSA total	65.4 ± 20.8	72.5 ± 17.6	*t*_(df = 97)_ = −1.8 *p = 0.07*	*F*_(df = 1,96)_ = 11.1 ***p* < 0.001**	*F*_(df = 1,96)=_3.4 *p = 0.06*
PSP total	44.8 ± 14.3	51.5 ± 12.9	*t*_(df = 97)_ = −2.4 ***p* = 0.02**	*F*_(df = 1,96)_ = 9.01 ***p* = 0.003**	*F*_(df = 1,96)_ = 6.01 ***p* = 0.016**
SLOF Area 5—Community living	40.8 ± 6.8	45.8 ± 5.8	*t*_(df = 87)_ = 3.7 ***p* < 0.0001**	*F*_(df = 1,86)_ = 16.3 ***p* < 0.0001**	*F*_(df = 1,86)_ = 13.6 ***p* < 0.0001**

*Note:* The table describes means and SD (rates for gender) of demographic and clinical factors in the groups of early onset (EOS) and adult onset (AOS) schizophrenia patients. Significant differences have been highlighted in bold. Trends toward significance (*p* > 0.05 and <0.08) were given in italics. Additional significant results are given in Table S1.

Abbreviations: AaFP, age at first psychiatric contact; NES, Neurological Examination Scale; PANSS, Positive and Negative Syndrome Scale; PSP, Personal and Social Performance scale; SLOF, Specific Level of Functioning scale; UPSA, University of San Diego Performance‐based Skills Assessment scale.

^a^ Adjustment for age.

^b^ Adjustment for duration of illness.

No significant differences between AOS and EOS patients were found in number of hospitalizations or mean daily antipsychotic doses, even after adjustment for age or duration of illness (Table S1). On the contrary, AOS patients showed a trend toward significance for higher number of compulsory hospitalizations. However, this result did not survive adjustment for age and it may simply depend on higher mean age in AOS (Table S1). Disease severity, as assessed by CGI‐S, was not significantly different between the two groups. Nonetheless, after adjustment for age, a trend toward significance was found (Table S1), with adjusted estimated means showing a sharper divergence between EOS and AOS (4.35 ± 0.11 vs. 4.02 ± 0.11, respectively).

EOS patients had significantly more severe psychotic symptoms. Indeed, EOS patients exhibited higher mean scores on PANSS total, PANSS general psychopathology scale, and PANSS 5‐factor Disorganization factor (Table 1). All of these significant differences survived adjustment for both age and duration of illness (Table 1). Significantly higher mean scores in EOS were also observed for the Positive and Excitement factors; however, these significances did not survive adjustment for age (Table S1, note that for the Excitement factor, a trend toward significance was found after adjustment for age).

Cognitive functioning was more impaired in EOS patients, although results were controversial. EOS patients had lower mean scores on the VSM, a result that survived adjustment for age and for duration of illness (Table 1). A trend toward significance was also found for more impaired verbal memory performances in EOS compared to AOS. This trend remained even after adjustment for age or for duration of illness (Table S1). A trend toward significance for more impaired working memory performances was also found after adjustment for age (Table S1). According to the more severe cognitive impairment in EOS patients, neurological soft signs were more obvious in this group. Notably, although NES score was not significantly different between groups at the Student's *t* test, a clear significant difference emerged after adjustment for age (Table 1). A similar pattern was also observed for the sensory integration and other signs NES subscales, whose significant differences emerged after adjustment for age (Table S1). In all cases, the extent of neurological soft signs was larger in EOS compared to AOS, which is consistent with a more neurodevelopmentally based origin of the disease in those who develop frank symptoms in an earlier age.

According to the results in clinical variables, EOS patients were more impaired in social functioning. Indeed, EOS patients had significantly lower scores on the UPSA, the PSP, and at least in the SLOF Area5, that measures community living functioning. All these significant differences survived adjustment for age (Table 1). Notably, the significant difference on the UPSA emerged after adjustment for age (Table 1).

These results were confirmed by multiple regression models with age at onset (continuous) as the independent variable, even when adjusted for age and duration of illness (Table S2).

### 3.2 Relationships between age at onset and response to antipsychotics

We evaluated whether EOS were more frequently diagnosed as TRS compared to AOS. Although only a trend toward significance was found (*χ* = 3.01, df = 1, *p* = 0.081), TRS patients had significantly lower age‐at‐onset than nonTRS, after adjustment for age (ANCOVA, *F*_1,96_ = 11.32, *p* = 0.001).

Consistently with this result, we subdivided the sample into four groups (i.e., EOS‐TRS; EOS‐nonTRS; AOS‐TRS; AOS‐nonTRS) and compared them on multiple clinical, cognitive, and psychosocial variables, including age as a covariate. We expected that the EOS‐TRS would be the most impaired group. Given the epidemiological observation that male individuals may have earlier age of psychosis onset than female ones (Li et al., 2016), we also controlled for gender effect by two‐way ANCOVA. Briefly, the interaction between gender and age at onset was significant only for age at first psychiatric evaluation (however, entirely driven by the age at onset effect). A significant, albeit weak, significant interaction was found for antipsychotic doses (as reported in more details below). No significant interaction was found in all the other analyses, and either a significant age at onset independent effect or no independent effect were observed. Therefore, with the possible exclusion of antipsychotic doses, the other significant differences found herein did not seem to be driven by the male gender. All the outcomes of these analyses are available upon request.

### 3.3 Clinical variables

EOS‐TRS were under significantly higher antipsychotic doses, had longer duration of disease, earlier age at first psychiatric contact, and higher disease severity compared to other groups (Table 2). Other significant inter‐group differences were reported in Table S3. Notably, a weak significant interaction was found between gender and age‐at‐onset (*F*_3,90_ = 2.6, *p* = 0.05), with both independent effects that were significant (Gender: *F*_1,90_ = 8.12, *p* = 0.05; age‐at‐onset: *F*_3,90_ = 2.77, *p* = 0.46). The highest mean antipsychotic doses were prescribed to male early onset patients, both TRS and non‐TRS.

**TABLE 2:** Clinical variables' outcomes

Variable	EOS‐TRS^a^ (*n* = 31)	EOS‐nonTRS^a^ (*n* = 22)	AOS‐TRS^a^ (*n* = 18)	AOS‐nonTRS^a^ (*n* = 28)	Statistics^b^	Multiple comparisons^c^
Antipsychotic doses	598.42 ± 49.71	349.18 ± 64.85	559.28 ± 68.48	345.75 ± 53.65	*F*_(df = 3,94)_ = 6.09 *p* = 0.001	EOS‐TRS > nonTRS (EOS and AOS) AOS‐TRS > nonTRS (EOS and AOS)
Duration of disease	19.6 ± 0.81	18.13 ± 1.05	11.33 ± 1.11	9.9 ± 0.87	*F*_(df = 3,94)_ = 25.1 *p* < 0.0005	EOS‐TRS > AOS (TRS and nonTRS) EOS‐nonTRS > AOS (TRS and nonTRS)
Age at first psychiatric evaluation	18.71 ± 0.88	21.17 ± 1.15	25.67 ± 1.22	27.89 ± 0.95	*F*_(df = 3,94)_ = 17.1 *p* < 0.0005	EOS‐TRS < AOS (TRS and nonTRS) EOS‐nonTRS < AOS (TRS and nonTRS)
CGI‐S	4.5 ± 0.1	3.9 ± 0.1	4.3 ± 0.1	3.8 ± 0.1	*F*_(df = 3,91)_ = 5.8 *p* = 0.001	EOS‐TRS > nonTRS (EOS and AOS) AOS‐TRS > AOS‐nonTRS
PANSS total	99.69 ± 2.77	85.37 ± 3.62	91.51 ± 3.82	83.74 ± 2.99	*F*_(df = 3,94)_ = 6.3 *p* = 0.001	EOS‐TRS > nonTRS (EOS and AOS)
NES total	25.37 ± 1.74	16.14 ± 2.27	17.11 ± 2.4	17.23 ± 1.88	*F*_(df = 3,94)_ = 5.57 *p* = 0.001	EOS‐TRS > all groups
Working memory	0.43 ± 0.21	1.27 ± 0.25	0.81 ± 0.27	1.41 ± 0.21	*F*_(df = 3,92)_ = 4.45 *p* = 0.006	EOS‐TRS < nonTRS (EOS and AOS)
Verbal memory	0.89 ± 0.27	1.72 ± 0.34	1.83 ± 0.37	1.78 ± 0.29	*F*_(*df = 3*,*92*)_ *= 2.35* *p = 0.07*	EOS‐TRS < AOS‐nonTRS
Visuospatial memory	22.78 ± 2.97	33.48 ± 4.19	36.31 ± 3.95	35.66 ± 3.2	*F*_(df = 3,69)_ = 3.96 *p* = 0.011	EOS‐TRS < all groups
UPSA total	57.87 ± 3.24	69.48 ± 4.23	78.6 ± 4.47	73.62 ± 3.5	*F*_(df = 3,94)_ = 5.81 *p* = 0.001	EOS‐TRS < all groups
PSP total	40.09 ± 2.32	50.16 ± 3.03	46.91 ± 3.2	55.5 ± 2.51	*F*_(df = 3,94)_ = 7.21 *p* < 0.0005	EOS‐TRS < nonTRS (EOS and AOS) AOS‐TRS < AOS‐nonTRS
SLOF Area5 Community living	38.24 ± 1.1	44.16 ± 1.46	43.18 ± 1.47	47.79 ± 1.19	*F*_(df = 3,84)_ = 11.9 *p* < 0.0005	EOS‐TRS < all groups AOS‐TRS < AOS‐nonTRS

*Note:* The table describes means and SE of clinical and psychopathological factors in EOS (early onset)–TRS (treatment resistant schizophrenia), EOS‐nonTRS, AOS (adult onset)‐TRS, and AOS‐nonTRS patients. Significant differences have been highlighted in bold. Additional significant differences were given in Table S3.

Abbreviations: CGI‐S, Clinical Global Impression‐Severity scale; NES, Neurological Examination Scale; PANSS, Positive and Negative Syndrome Scale; PSP, Personal and Social Performance scale; SLOF, Specific Level of Functioning scale; UPSA, University of San Diego Performance‐based Skills Assessment scale.

^a^ All means were adjusted for age.

^b^ ANCOVA with age as a covariate.

^c^ Least‐square difference.

At the two‐way ANOVA, a significant TRS status effect for antipsychotic doses and CGI‐S score was found. A significant age‐at‐onset effect was found for the duration of disease, while significant differences in age at first psychiatric contact derived from independent TRS status and age‐at onset effects (Table 3).

**TABLE 3:** Outcomes of two‐way ANOVA

Variable	Age at onset effect^a^		TRS status effect^a^		Combined effect^a^	
	*F*_(df)_	*p*	*F*_(df)_	*p*	*F*_(df)_	*p*
Antipsychotic dose	0.97_(1,95)_	>0.05	**16.52**_**(1,92)**_	**<0.0005**	0.174_(1,92)_	>0.05
Duration of disease	**59.32**_**(1,94)**_	**<0.0005**	2.62_(1,94)_	>0.05	0.014_(1,94)_	>0.05
AaFP	**34.02**_**(1,94)**_	**<0.0005**	**5.27**_**(1,94)**_	**0.024**	0.02_(1,94)_	>0.05
CGI‐S	0.78_(1,91)_	>0.05	**11.36**_**(1,91)**_	**0.001**	0.0005_(1,91)_	>0.05
PANSS total score	1.95_(1,94)_	>0.05	**10.22**_**(1,94)**_	**0.002**	0.77_(1,94)_	>0.05
PANSS Positive	0.34_(1,94)_	>0.05	**6.49**_**(1,94)**_	**0.012**	0.35_(1,94)_	>0.05
PANSS Negative	1.15_(1,94)_	>0.05	**3.99**_**(1,94)**_	**0.048**	0.63_(1,94)_	>0.05
PANSS general psychopathology	2.32_(1,94)_	>0.05	**10.76**_**(1,94)**_	**0.001**	1.71_(1,94)_	>0.05
Positive factor	0.47_(1,89)_	>0.05	**7.61**_**(1,89)**_	**0.007**	0.13_(1,89)_	>0.05
Disorganization factor	*3.21*_(*1*,*89*)_	*0.07*	**7.42**_**(1,89)**_	**0.008**	2.09_(1,89)_	>0.05
Excitement factor	0.77_(1,89)_	>0.05	**8.48**_(1,89)_	**0.005**	0.66_(1,89)_	>0.05
Emotional distress factor	0.69_(1,89)_	>0.05	**8.37**_**(1,89)**_	**0.005**	0.31_(1,89)_	>0.05
Working memory	0.96_(1,92)_	>0.05	**9.41**_**(1,92)**_	**0.003**	0.28_(1,92)_	>0.05
Visuospatial memory	**3.96**_**(1,69)**_	**0.05**	2.5_(1,69)_	>0.05	*3.26*_(*1*,*69*)_	*0.07*
NES total score	2.68_(1,94)_	>0.05	**3.84**_**(1,94)**_	**0.05**	**4.35**_**(1,94)**_	**0.04**
NES sensory integration	1.82_(1,91)_	>0.05	2.58_(1,92)_	>0.05	***6.42***_**(*1*,*91*)**_	***0.013***
NES other signs	1.84_(1,91)_	>0.05	**6.43**_**(1,91)**_	**0.013**	2.13_(1,91)_	>0.05
UPSA total score	**8.96**_**(1,94)**_	**0.004**	0.27_(1,94)_	>0.05	*3.51*_(*1*,*94*)_	*0.06*
PSP total score	**4.08**_**(1,94)**_	**0.046**	**10.73**_**(1,94)**_	**0.001**	0.3_(1,94)_	>0.05
SLOF Area 5	**8.96**_**(1,84)**_	**0.004**	**17.38**_**(1,84)**_	**<0.0005**	0.37_(1,84)_	>0.05

*Note:* The table reports the outcomes of two‐way ANOVA with TRS/non‐TRS status and EOS/AOS age at onset as the independent categorical variables. As the dependent variables we included all those resulting significant different among groups at the ANCOVA analysis. Significant values were given in bold. Trend toward significance (*p* > 0.05 and <0.08) was given in italics. Combined effects without significance of one or both independent effects were given in bold italics.

Abbreviation: AaFP, age at first psychiatric evaluation.

^a^ Adjusted for age.

### 3.4 Symptom variables

PANSS total score was significantly higher in EOS‐TRS compared to nonTRS patients (Tables 2 and S3). In all PANSS subscale scores, EOS‐TRS showed significantly higher scores.

When using the 5‐Factor PANSS subdivision (van der Gaag et al., 2006), we found significant higher mean scores in EOS‐TRS compared to nonTRS groups in Positive, Excitement, and Emotional Distress factors and compared to all groups in the Disorganization factor (Tables 2 and S3).

In all cases, significant differences among groups were explained by TRS status effect at the two‐way ANOVA (Table 3). A trend toward significance for an age‐at‐onset effect was found for the Disorganization factor.

### 3.5 Cognitive variables and NSS

Compared to all the other groups, EOS‐TRS exhibited higher NES total and sensory integration scores, and higher Other Signs scores compared to nonTRS groups (Tables 2 and S3).

EOS‐TRS had significantly worse VSM compared to all groups, worse working memory performances compared to nonTRS groups, and a trend toward significance for worse verbal memory performances (Tables 2 and S3).

The two‐way ANOVA revealed that the significant differences among groups in working memory depended on TRS status effect, while an age‐at‐onset effect and a trend toward significance for combined effect explained the differences in VSM (Table 3). Significant differences in NES Total score depended on TRS status and combined effects, TRS status effect explained differences in Other Signs scores, combined effects but no independent effect were found for sensory integration (Table 3).

### 3.6 Social functioning variables

PSP total and UPSA total score was significantly lower in EOS‐TRS compared to all other groups. PSP score was significantly lower in EOS‐TRS compared to nonTRS groups (Table 2). Among SLOF subscales, EOS‐TRS performed significantly worse than all other groups on SLOF Area5 (Table 2).

At the two‐way ANOVA, significant differences among groups at the UPSA were attributable to an age‐at‐onset effect and a trend toward significance combined effect (Table 3). Independent TRS status and age‐at‐onset effects were found for PSD and SLOF Area5 (Table 3).

### 3.7 Alternative PANSS Positive‐based definition of TRS

Analyses were also run using the alternative PANSS‐based definition of TRS. The rate of TRSp was significantly higher in EOS, and the rate of nonTRSp was significantly higher in AOS (*χ* = 6.31, df = 1, *p* = 0.012). Notably, the distribution of patients within the TRS/nonTRS categories changed, after having adopted this alternative definition. Although the two definitions were correlated (Spearman's rho: *p* = 0.002; rho = 0.31), the distribution of patients was significantly different (Chi‐square: *p* = 0.002; *χ* = 9.42).

Despite some minor differences, outcomes at the ANCOVA and two‐way ANOVA were similar to those found with the traditional TRS definition. However, effect sizes were larger (Tables S4–S6). In our opinion, the PANSS Positive‐based alternative definition may better outline antipsychotic refractoriness and should be used in place of the traditional, PANSS total‐based TRS definition.

## 4 DISCUSSION

The present study aimed at analysing the relationship between the condition of non‐response to antipsychotic agents and the age at onset of the first psychotic episode in schizophrenia patients. Previous studies have reported that earlier age at onset is associated with a more severe course of the illness, putatively as an effect of unique psychopathology and neurobiology (Clemmensen et al., 2012; Rabinowitz et al., 2006; Sato et al., 2004).

Accordingly, in our sample we found that EOS patients exhibited more severe clinical, cognitive, and psychosocial outcomes. Notably, early onset patients also exhibited a higher severity of neurological soft signs, which are considered proxy measures of aberrant neurodevelopment (Hirjak et al., 2019). Differences between groups were driven, at least in part, by male gender only in the case of mean antipsychotic doses, a finding that may be reasonably attributed to more alarming clinical presentations in males, for example, aggressiveness or restlessness, rather than to a greater severity of the disease.

We also found that the rates of TRS patients in early onset individuals was significantly higher than non‐TRS ones. Consistently, TRS patients as a group had a significantly earlier mean age at onset than non‐TRS ones. Notably, TRS has been considered a potential subtype of schizophrenia, with its putative neurobiology and clinical course (de Bartolomeis et al., 2018; Demjaha et al., 2012; Nucifora et al., 2019). Therefore, we investigated the hypothesis that early onset TRS patients may have more severe clinical presentation compared to the other groups of patients, even including TRS ones with adult onset of psychotic symptoms.

According to our hypothesis, we found that the group with the most impaired clinical variables was that of the early onset TRS patients. Intriguingly, most of these variables have been extensively associated with the key neurobiological processes of schizophrenia (Adhikari et al., 2019; Broome et al., 2010; Collin et al., 2016; Gillespie et al., 2017), leading to conceptual disorganization, formal thought disturbances, and other cognitive impairments (Stephan et al., 2009).

Overall, the earlier age at onset in TRS patients is in agreement with the view that TRS may be categorically distinct from treatment‐responsive schizophrenia (Gillespie et al., 2017), and it may suggest a more neurodevelopmentally oriented pathophysiologic process. Our data may represent a clinical support to the view that common neurobiological backgrounds may be responsible for both earlier presentation of psychotic symptoms and lack of response to antipsychotic agents.

However, the significant impairment in clinical outcomes may be mostly associated with being non‐responder to antipsychotic agents rather than with the earlier onset of psychotic symptoms. To verify this hypothesis, we carried out a series of two‐way ANOVA with age at onset and TRS status as the independent variables. These analyses showed that a significant independent effect of age at onset might be found only for a small number of variables.

On the contrary, in almost all variables in which differences among groups were found, those differences might be attributed to a TRS status rather than to an age at onset effect. This result may suggest that early onset TRS patients were more impaired and clinically severe than the other schizophrenia groups due to the condition of non‐response to antipsychotics, which is regarded to be a proxy of a distinct, more severe neurobiological subtype of schizophrenia (Leung et al., 2019; Nucifora et al., 2019). Therefore, early onset TRS patients may suffer from a distinct neurobiological entity compared to adult onset TRS and responder schizophrenia patients. These data comply with the neurodevelopmental hypothesis of schizophrenia, which has recently received substantial support from the observation that genetic variants associated with schizophrenia converge on a developmental trajectory sensitive to events that affect the placental response to stress (Ursini et al., 2018). Clinical severity may be heightened from early onset, thus explaining the significant differences even with adult onset TRS. Likewise, the higher severity in early onset compared to AOS patients may thus be in part explained by higher rates of TRS patients in the early onset group, due to the peculiar neurobiological base of this condition.

These findings imply that conspicuous efforts should be provided to improve diagnostic sensibility in the early stages of psychosis, possibly deeming increased attention to subtle psychopathological manifestations that had occurred during paediatric age. These manifestations may be suggestive of neurodevelopmentally based alterations showing their effects even before prodromal signs of psychosis during adolescence (Dolz et al., 2019). These procedures may help to promote early intervention in at‐risk individuals. Moreover, given the observation that early onset may associate with treatment resistance, patients with early onset of psychotic symptoms should be strictly and rigorously monitored for their response to antipsychotics, and switch to clozapine should be attempted early in the course of the disease.

Nonetheless, the hypothesis that early onset TRS patients may suffer from a distinct neurobiological entity needs to be deepened in broader samples of patients and it may also serve to contribute to the attempts of reconceptualizing the construct of clinical high risk of psychosis that has been recently criticized (Raballo & Poletti, 2019).

The results of the present study should be discussed considering its limitations. The study has a cross‐sectional design, which prevents from evaluating the longitudinal course of the disease from its onset. Longitudinal studies are cost‐expensive and often require multicenter approaches, which diminish the homogeneity of the sample. However, a longitudinal design would be warranted to confirm and expand the results of our study. We missed to include Duration of Untreated Psychosis among the variables taken into account, as we were able to retrieve records of the first effective antipsychotic treatment since psychosis onset in only a small minority of patients. However, including this variable would have provided additional information on the role of early onset, and possibly delay in initiating treatments, for subsequent clinical outcomes.

Although the overall sample resulted in 99 patients out of 197 screened, the sample should be evaluated based on the stringent inclusion and exclusion criteria used herein, which we consider mandatory to delineate a reliable sample of non‐affective psychotic subjects. Also, TRS patients are considered a minority of schizophrenia patients and thus a representative sample is considered to be at least 10%–30% of a representative schizophrenia sample (Kane et al., 2019).

It should be noted that, in our sample, patients defined as TRS were approximately one half of the included sample of schizophrenia patients, a proportion that is higher than in previous reports (Assunção‐Leme et al., 2020; Chan et al., 2020; Teo et al., 2012). Moreover, both TRS and nonTRS patients had considerably high mean PANSS scores, indicating high severity of psychotic symptoms also in those who were found to respond to antipsychotic agents. This peculiarity is to be attributed to our academic unit's unique organization since patients who had been found partially or no responders to usual treatments in community settings were referred to our unit for a more structured diagnosis and potential management of TRS. This peculiar modality of referral contributed to selecting a sample of severe patients with a high proportion of treatment resistant ones. Nonetheless, the similar proportion of TRS and nonTRS patients and their comparable and severe clinical conditions allowed, in our opinion, a less biased comparison between these groups than in other reports.

In conclusion, the results showed herein may suggest that previous studies reporting an association between age at onset and worse clinical outcomes might have also measured an indirect effect, whose primary determinant could be the poor response to antipsychotics. This, in turn, could depend on multiple neurobiological alterations, whose higher severity compared to the ones present in responder patients may lead to an earlier onset of psychotic symptoms.

## CONFLICT OF INTEREST

All authors disclose any actual or potential conflict of interest including any financial, personal or other relationships with other people or organizations within 3 years of beginning the work submitted that could inappropriately influence, or be perceived to influence, their work.

## Supporting information


**Appendix** **S1:** Supporting information

## Data Availability

All the data in the present study are available on request due to privacy/ethical restrictions
