# Supplementary material for: Relationships between early age at onset of psychotic symptoms and treatment resistant schizophrenia
Source: Early Interv Psychiatry. 2021 May 16;16(4):352–62. doi: 10.1111/eip.13174 (PMC9291026; doi:10.1111/eip.13174)
Supplement: Supplementary file 1 — Appendix S1: Supporting information [file EIP-16-352-s001.pdf]

**Variable**	**EOS (n=53)**	**AOS (n=46)**	**Statistics**	**Adjusted Statistics[a]**	**Adjusted Statistics[b]**
Hospitalizations	1.3 ± 1.5	1.9 ± 3.4	$t_{(df=95)}$=-1.1 p=0.27	$F_{(df=1,94)}$=0.05 p=0.81	$F_{(df=1,94)}$=1.31 p=0.25
Compulsory Hospitalizations	0.2 ± 0.6	0.6 ± 1.3	$t_{(df=96)}$=-1.8 *p=0.07*	$F_{(df=1,95)}$=1.3 p=0.25	*$F_{(df=1,95)}$=3.5 p=0.06*
Antipsychotic Doses	507.6 ± 327.7	414.7 ± 247.1	$t_{(df=97)}$=1.57 p=0.11	$F_{(df=1,96)}$=2.2 p=0.14	$F_{(df=1,96)}$=2.4 p=0.12
CGI-S	4.3 ± 0.9	4.1 ± 0.6	$t_{(df=94)}$=1.7 p=0.102	$F_{(df=1,93)}$=3.8 *p=0.06*	$F_{(df=1,93)}$=2.6 p=0.11
Positive Factor	24.4 ± 7.2	21.6 ± 7.7	$t_{(df=92)}$=2.1 **p=0.04**	$F_{(df=1,91)}$=2.5 p=0.11	$F_{(df=1,91)}$=4.3 **p=0.04**
Excitement Factor	21.9 ± 5.6	19.5 ± 4.5	$t_{(df=92)}$=2.3 **p=0.03**	$F_{(df=1,91)}$=3.4 *p=0.07*	$F_{(df=1,91)}$=4.8 **p=0.03**
NES Sensory Integration	4.3 ± 2.9	3.6 ± 2.4	$t_{(df=94)}$=1.3 p=0.21	$F_{(df=1,93)}$=4.0 **p=0.05**	$F_{(df=1,93)}$=1.6 p=0.21
NES Other Signs	8.8 ± 5.1	7.7 ± 3.9	$t_{(df=94)}$=1.2 p=0.24	$F_{(df=1,93)}$=5.2 **p=0.03**	$F_{(df=1,93)}$=1.2 p=0.25
Verbal Memory	1.25 ± 1.6	1.8 ± 1.4	$t_{(df95=)}$=-1.8 *p=0.07*	$F_{(df=1,94)}$=3.3 *p=0.07*	$F_{(df=1,94)}$=3.3 *p=0.07*
Working Memory	0.82 ± 1.2	1.15 ± 1.13	$t_{(df95=)}$=-1.4 p=0.16	$F_{(df=1,94)}$=3.4 *p=0.07*	$F_{(df=1,94)}$=2.1 p=0.15

Supplementary Table 1. Demographic and clinical factors. The table describes means and standard deviation (rates for gender) of demographic and clinical factors in the groups of early onset (EOS) and adult onset (AOS) schizophrenia patients. Significant differences have been highlighted in bold. Trends toward significance (p>0.05 and <0.08) were given in italics. [a]: adjustment for age. [b]: adjustment for duration of illness. CGI-S: Clinical Global Impression-Severity. NES: Neurological Evaluation Scale.

	Model 1	Model 2	Model 3
Age	**<.0005**		**<.0005**
Years of Schooling	**ns**	**ns**	**ns**
Hospitalizations	**ns**	**ns**	**ns**
Compulsory Hospitalizations	**ns**	**ns**	ns
AaFP	**<.0005**	**<.0005**	**<.0005**
Duration of Illness	**ns**	**<.0005**	
CPZ	**ns**	**ns**	**ns**
CGI-S	**ns**	**ns**	**ns**
PANSS total	ns	ns	ns
PANSS-GP	**.021**	**.006**	**.033**
POS	**.036**	**ns**	**.033**
DIS	ns	**.019**	ns
EXC	**.007**	***.018***	**.008**
NEStotal	**ns**	***.07***	**ns**
NES-SI	**ns**	**.006**	**ns**
NES-OS	**ns**	***.07***	**ns**
Verbal Memory	*.07*	***.06***	ns
Working Memory	.013	**.001**	.022
VSM	**.05**	**.013**	***.07***
PSPTotal	**.05**	**.01**	ns
UPSATotal	ns	**.004**	ns
SLOFArea5	**.002**	**<.0005**	**.003**

Supplementary Table 2. Regression model with age at onset as a continuous variable. We performed three different models of linear regression (model 1: age at onset as the independent variable (IV); model 2: age at onset + age IVs; model 3: age at onset + duration of illness IVs). Reported values indicated whether in each model age at onset was a significant predictor of the variable indicated in the left column. ns: not significant.

**Variable**	**EOS-TRS[a]**	**EOS-nonTRS[a]**	**AOS-TRS[a]**	**AOS-nonTRS[a]**	**Statistics[b]**	**Multiple Comparisons[c]**
PANSS Positive	21.89 ± 0.91	18.11 ± 1.18	20.46 ± 1.24	18.32 ± 0.97	$F_{(df=3,94)}=3.5$ $p=0.01$	EOS-TRS > nonTRS (EOS and AOS)
PANSS Negative	25.24 ± 0.94	22.91 ± 1.23	24.08 ± 1.3	21.55 ± 1.01	$F_{(df=3,94)}=2.5$ $p=0.05$	EOS-TRS > AOS-nonTRS
PANSS General Psychopathology	52.53 ± 1.53	44.13 ± 1.99	47.13 ± 2.11	43.48 ± 1.65	$F_{(df=3,94)}=6.81$ $p<0.0005$	EOS-TRS > all groups
Positive Factor	25.77 ± 1.15	21.15 ± 1.56	24.16 ± 1.62	20.77 ± 1.28	$F_{(df=3,89)}=3.74$ $p=0.014$	EOS-TRS > nonTRS (EOS and AOS)
Disorganization Factor	34.21 ± 1.15	28.32 ± 1.56	29.43 ± 1.62	27.6 ± 1.28	$F_{(df=3,89)}=6.15$ $p=0.001$	EOS-TRS > all groups
Excitement Factor	23.11 ± 0.89	19.14 ± 1.21	21.23 ± 1.25	18.94 ± 0.99	$F_{(df=3,89)}=4.34$ $p=0.007$	EOS-TRS > nonTRS (EOS and AOS)
Emotional Distress Factor	28.08 ± 1.04	23.53 ± 1.42	26.99 ± 1.47	23.99 ± 1.17	$F_{(df=3,89)}=3.66$ $p=0.015$	EOS-TRS > nonTRS (EOS and AOS)
NES Sensory Integration	5.34 ± 0.45	3.09 ± 0.62	3.16 ± 0.62	3.64 ± 0.5	$F_{(df=3,91)}=4.58$ $p=0.005$	EOS-TRS > all groups
NES Other Signs	10.53 ± 0.78	7.06 ± 1.06	7.86 ± 1.07	6.83 ± 0.86	$F_{(df=3,91)}=4.34$ $p=0.007$	EOS-TRS > nonTRS (EOS and AOS)

Supplementary Table 3. Clinical variables' outcomes. The table describes means and standard error of cognitive and psychosocial factors in EOS–TRS, EOS-nonTRS, AOS-TRS, and AOS-nonTRS patients. Significant differences have been highlighted in bold. Italics was used to denote trend toward significance. [a]: all means were adjusted for age. [b]: ANCOVA with age as a covariate. [c]: Least-Square Difference. PANSS: Positive and Negative Syndrome scale. NES: Neurological Examination Scale.

Variable	EOS-TRSp[a]	EOS-nonTRSp[a]	AOS-TRSp[a]	AOS-nonTRSp[a]	Statistics[b]	Multiple Comparisons[c]
Antipsychotic doses	567.97 ± 47.59	338.23 ± 78.12	470.85 ± 64.64	364.02 ± 59.37	$F_{(df=3,94)}=3.5$ p=0.018	EOS-TRSp > nonTRSp (EOS and AOS)
Duration of Disease	19.52 ± 0.74	17.74 ± 1.22	11.09 ± 1.01	9.75 ± 0.93	$F_{(df=3,94)}=25.3$ p<0.0005	EOS-TRSp > AOS (TRSp and nonTRSp)<br><br>EOS-nonTRSp > AOS (TRSp and nonTRSp)
Age at First Psychiatric Evaluation	18.78 ± 0.82	21.94 ± 1,34	26.88 ± 1.11	27.61 ± 1.02	$F_{(df=3,94)}=16.9$ p<0.0005	EOS-TRSp < all groups<br><br>EOS-nonTRSp < AOS (TRSp and nonTRSp)
CGI-S	4.6 ± 0.1	3.5 ± 0.1	4.3 ± 0.1	3.7 ± 0.1	$F_{(df=3,94)}=13.8$ p<0.0005	EOS-TRSp > nonTRSp (EOS and AOS)<br><br>AOS-TRSp > nonTRSp (EOS and AOS)
PANSS Total	101.04 ± 2.18	74.93 ± 3.58	93.66 ± 2.96	79.61 ± 2.72	$F_{(df=3,94)}=20.8$ p<0.0005	EOS-TRSp > nonTRSp (EOS and AOS)<br><br>AOS-TRSp > nonTRSp (EOS and AOS)
PANSS Positive	22.45 ± 0.71	14.71 ± 1.17	21.46 ± 0.97	16.87 ± 0.89	$F_{(df=3,94)}=16.3$ p<0.0005	EOS-TRSp > nonTRSp (EOS and AOS)<br><br>AOS-TRSp > nonTRSp (EOS and AOS)
PANSS Negative	25.59 ± 0.85	20.95 ± 1.39	23.41 ± 1.15	21.55 ± 1.06	$F_{(df=3,94)}=4.3$ p=0.006	EOS-TRSp > nonTRSp (EOS and AOS)
PANSS General Psychopathology	52.94 ± 1.21	39.04 ± 1.99	48.92 ± 1.65	40.74 ± 1.51	$F_{(df=3,94)}=20.2$ p<0.0005	EOS-TRSp > nonTRSp (EOS and AOS)<br><br>AOS-TRSp > nonTRSp (EOS and AOS)
Positive Factor	26.41 ± 0.8	16.13 ± 1.49	26.49 ± 1.22	18.24 ± 1.07	$F_{(df=3,89)}=22.9$ p<0.0005	EOS-TRSp > nonTRSp (EOS and AOS)<br><br>AOS-TRSp > nonTRSp (EOS and AOS)
Negative Factor	28.21 ± 1.05	22.08 ± 1.84	26.59 ± 1.51	22.82 ± 1.32	$F_{(df=3,89)}=5.07$ p=0.003	EOS-TRSp > nonTRSp (EOS and AOS)
Disorganization Factor	34.67 ± 0.93	23.67 ± 1.63	30.18 ± 1.33	26.31 ± 1.17	$F_{(df=3,89)}=17.2$ p<0.0005	EOS-TRSp < all groups<br><br>AOS-TRSp > nonTRSp (EOS and AOS)
Excitement Factor	23.31 ± 0.74	16.28 ± 1.31	21.85 ± 1.07	17.87 ± 0.94	$F_{(df=3,89)}=11.8$ p<0.0005	EOS-TRSp > nonTRSp (EOS and AOS)<br><br>AOS-TRSp > nonTRSp (EOS and AOS)
Emotional Distress Factor	27.83 ± 0.88	21.62 ± 1.55	28.58 ± 1.27	22.06 ± 1.12	$F_{(df=3,89)}=10.02$ p<0.0005	EOS-TRSp > nonTRSp (EOS and AOS)<br><br>AOS-TRSp > nonTRSp (EOS and AOS)

Supplementary Table 4. Clinical and psychopathological variables' outcomes. The table describes means and standard error of clinical and psychopathological factors in EOS (early onset)–TRSp (Treatment Resistant Schizophrenia), EOS-nonTRSp, AOS (adult onset)-TRSp, and AOS-nonTRSp patients. Significant differences have been highlighted in bold. [a]: all means were adjusted for age. [b]: ANCOVA with age as a covariate. [c]: Least-Square Difference. CGI-S: Clinical Global Impression-Severity scale. PANSS: Positive and Negative Syndrome scale.

**Variable**	**EOS-TRSp[a]**	**EOS-nonTRSp[a]**	**AOS-TRSp[a]**	**AOS-nonTRSp[a]**	**Statistics[b]**	**Multiple Comparisons[c]**
NES Total	23.26 ± 1.67	18.34 ± 2.75	16.97 ± 2.28	16.46 ± 2.09	$F_{(df=3,94)}=2.6$<br>$p=0.05$	EOS-TRSp > AOS (TRSp and nonTRSp)
NES Sensory Integration	4.99 ± 0.43	3.32 ± 0.73	2.75 ± 0.58	3.88 ± 0.54	$F_{(df=3,91)}=3.5$<br>$p=0.019$	EOS-TRSp > EOS-nonTRSp and AOS-TRSp
NES<br>Other Signs	9.66 ± 0.75	8.28 ± 1.27	7.84 ± 1.01	6.36 ± 0.94	*$F_{(df=3,91)}=2.3$*<br>*$p=0.07$*	EOS-TRSp > AOS-nonTRSp
VisuoSpatial Memory	24.61 ± 2.85	31.29 ± 4.95	34.97 ± 3.55	37.86 ± 3.61	$F_{(df=3,69)}=3.04$<br>$p=0.034$	EOS-TRSp < AOS (TRSp and nonTRSp)
Verbal Fluency	0.72 ± 0.19	1.26 ± 0.31	0.76 ± 0.25	1.44 ± 0.23	*$F_{(df=3,92)}=2.4$*<br>*$p=0.06$*	EOS-TRSp < AOS-nonTRSp<br><br>AOS-TRSp < AOS-nonTRSp
UPSA Total	58.99 ± 3.01	71.21 ± 4.94	77.56 ± 4.09	74.78 ± 3.76	$F_{(df=3,94)}=16.3$<br>$p<0.0005$	EOS-TRSp < all groups
PSP Total	40.61 ± 2.07	53.21 ± 3.41	46.71 ± 2.81	57.86 ± 2.58	$F_{(df=3,94)}=10.1$<br>$p<0.0005$	EOS-TRSp < nonTRSp (EOS and AOS)<br><br>AOS-TRSp < AOS-nonTRSp
SLOF Area5	39.07 ± 1.02	45.29 ± 1.9	44.67 ± 1.42	47.59 ± 1.28	$F_{(df=3,84)}=9.62$<br>$p<0.0005$	EOS-TRSp < all groups

Supplementary Table 5. Cognitive and psychosocial variables' outcomes. The table describes means and standard error of cognitive and psychosocial factors in EOS–TRSp, EOS-nonTRSp, AOS-TRSp, and AOS-nonTRSp patients. Significant differences have been highlighted in bold. Italics was used to denote trend toward significance. [a]: all means were adjusted for age. [b]: ANCOVA with age as a covariate. [c]: Least-Square Difference. NES: Neurological Examination Scale. UPSA: University of San Diego Performance-based Skills Assessment scale. PSP: Personal and Social Performance scale. SLOF: Specific Level of Functioning scale.

	Age at Onset Effect[a]		Diagnosis Effect[a]		Combined Effect[a]	
**Variable**	$F_{(df)}$	p	$F_{(df)}$	p	$F_{(df)}$	p
Antipsychotic Dose	$0.26_{(1,94)}$	>0.05	**$7.45_{(1,94)}$**	**0.008**	$1.004_{(1,94)}$	>0.05
Duration of Disease	**$57.62_{(1,94)}$**	**<0.0005**	$2.61_{(1,94)}$	>0.05	$0.52_{(1,94)}$	>0.05
AaFP	**$32.49_{(1,94)}$**	**<0.0005**	*$3.69_{(1,94)}$*	*0.058*	$1.12_{(1,94)}$	>0.05
CGI-S	$0.003_{(1,91)}$	>0.05	**$35.69_{(1,91)}$**	**<0.0005**	$2.28_{(1,91)}$	>0.05
PANSS Total Score	$0.18_{(1,94)}$	>0.05	**$50.58_{(1,94)}$**	**<0.0005**	***$4.61_{(1,94)}$***	***0.034***
PANSS Positive	$0.32_{(1,94)}$	>0.05	**$44.28_{(1,94)}$**	**<0.0005**	$2.92_{(1,94)}$	>0.05
PANSS Negative	$0.41_{(1,94)}$	>0.05	**$8.72_{(1,94)}$**	**0.004**	$1.61_{(1,94)}$	>0.05
PANSS General Psychopathology	$0.43_{(1,94)}$	>0.05	**$49.03_{(1,94)}$**	**<0.0005**	*$3.32_{(1,94)}$*	*0.07*
Positive Factor	$0.74_{(1,89)}$	>0.05	**$64.54_{(1,89)}$**	**<0.0005**	$0.77_{(1,89)}$	>0.05
Negative Factor	$0.07_{(1,89)}$	>0.05	**$12.06_{(1,89)}$**	**0.001**	$0.68_{(1,89)}$	>0.05
Disorganization Factor	$0.43_{(1,89)}$	>0.05	**$34.71_{(1,89)}$**	**<0.0005**	***$7.96_{(1,89)}$***	***0.006***
Excitement Factor	$0.004_{(1,89)}$	>0.05	**$29.58_{(1,89)}$**	**<0.0005**	$2.28_{(1,89)}$	>0.05
Emotional Distress Factor	$0.21_{(1,89)}$	>0.05	**$27.99_{(1,89)}$**	**<0.0005**	$0.017_{(1,89)}$	>0.05
Verbal Fluency	$0.15_{(1,92)}$	>0.05	**$6.08_{(1,92)}$**	**0.016**	$0.08_{(1,92)}$	>0.05
VisuoSpatial Memory	**$4.09_{(1,74)}$**	**0.047**	$1.86_{(1,74)}$	>0.05	$0.34_{(1,74)}$	>0.05
NES Total Score	$2.81_{(1,94)}$	0.09	$1.55_{(1,94)}$	>0.05	$1.03_{(1,94)}$	>0.05
NES Sensory Integration	$1.75_{(1,91)}$	>0.05	$0.22_{(1,91)}$	>0.05	***$6.14_{(1,91)}$***	***0.015***
NES Other Signs	$2.89_{(1,91)}$	0.09	$2.09_{(1,91)}$	>0.05	$0.003_{(1,91)}$	>0.05
UPSA Total Score	**$6.43_{(1,94)}$**	**0.013**	$1.45_{(1,94)}$	>0.05	***$3.72_{(1,94)}$***	***0.05***
PSP Total Score	$3.21_{(1,94)}$	>0.05	**$19.55_{(1,94)}$**	**<0.0005**	$0.07_{(1,94)}$	>0.05
SLOF Area 5	**$6.44_{(1,84)}$**	**0.013**	**$10.33_{(1,84)}$**	**0.002**	$1.35_{(1,84)}$	>0.05

Supplementary Table 6. Outcomes of 2way ANOVA. The table reports the outcomes of 2way ANOVA with TRSp/non-TRSp Diagnosis and EOS/AOS Age at Onset as the independent categorical variables. As the dependent variables we included all those resulting significant different among groups at the ANCOVA analysis. [a]: adjusted for age. Significant values were given in bold. Trend toward significance (p>0.05 and <0.08) was given in italics. Combined effects without significance of one or both independent effects were given in bold italics. AaFP: Age at First Psychiatric Evaluation.
